# Reflex Nervous Influence

**Published:** 1884-02

**Authors:** 


### SELECTIONS.

### MASSAGE.

The *London Med. Record*, June 5, 1883, says that Dr. Beuster, of Berlin, read a paper on this subject at a meeting of the Verein fur innere Medicin in Berlin, on January 8 (*Deutsche Med. Wochensch*, March 12 and 28th). It is desirable to consider the advantages of this form of treatment on nervous diseases as well as on others, in order to fix the limit of its operation. The treatment which is now being judged by the medical world is the oldest of all, and has been employed by men in every country and in every age. It was known to the Asclepiades, to Hippocrates and Galen, and the Greeks practiced it in the fourth century B. C. In Rome, in the time of Nero and Trajan, massage formed part of the programme carried out in the tepidarium of the public baths. The Brahmins in India practiced it under the name of shampooing, and Alexander the Great, when he was in India, allowed those of his soldiers who had been bitten by serpents to be so treated by the priests. It was brought to Germany by the Crusaders from Syria and Palestine, but it soon passed out of the physicians' hands into those of the people, and was employed merely as a pleasurable sensation. Travelers relate stories of its extension to every quarter of the globe. The natives of Nubia and Sennar employ it largely, and Professor Hartman found its beneficial effects during an attack of fever to be so decided that he regretted having refused so long to submit to it. Dr. Emerson tells of its employment by the Sandwich Islanders, under the name of Lomi-Lomi, and honored guests are there shampooed as a special mark of regard. In some cases, the people lay themselves on the ground and allow their children to run over their bodies by way of massage, a proceeding which is also seen in the province of Brandenberg. In Japan it has been used from the most ancient time as a refreshment after over-fatigue, as well as to cure diseases. Massage seems, however, to have been employed earliest of all by the Chinese. In the beginning of the present century a book, "Cong-Fou," 3,000 years old, was translated by the missionaries Hus and Amiot, in the medical part of which all the proceedings of the Swedish gymnastics are described so fully, as to render it likely that they were really taken from this work.

The following are the different movements of massage as now practiced, and as the French have formulated them.

1. *Effleurage.—Friction douce.*—Slow, gentle strokes in a centripetal direction along the course of the veins and lymphatics, made with the palm of the hand, oiled, and with the pressure intermitting, so as to cause passive peristaltic action.

2. *Massage a friction*, in which the finger-tips of one hand, held at right angles to the axis of the limb, rub across and across in narrow ellipses, while the fingers of the other hand stroke parallel to the axis of the limb from above downwards.

3. *Kneading* (*petrissage*), which should also be done from the periphery towards the centre, and which consists in raising up the soft parts and kneading them in a way that may be compared to squeezing a full sponge.

4. *Tapotement*, or tapping or striking, causing concussion of the affected part, which may be done with the fingers, the palm, the margin of the hand, the closed fist, a percussion-hammer, or an instrument like a drumstick, with an India rubber head and a whalebone stem.

The French have also various other instruments, and they employ also the passive movements of flexion, abduction, adduction, rotation, etc.

The amount of power required to be executed is very variable, and it wants much practice and experience. The number of sittings may vary from two to five in the day, and their duration from three to twenty minutes, or even one or two hours. The proceedings seem to cause the operator not only fatigue, but also a nervous excitability, from the action on the nerves of the fingers and hands.

The chief point of the question is the physiological effect which it has on the human body. Professor von Mosengeil thoroughly discussed the question in the German Surgical Congress in April, 1875. The centripetal stroking favors the venous and lymphatic circulation, and acts backwards even on the parts which are not touched, so that a greater quantity of blood passes through the parts, causing increased tissue-metamorphosis. It is clear that the formation of exudation will be thereby prevented, and that exudation already formed will be removed, and even more solid formations will be fattily degenerated, and so absorbed. Pathological products can be removed even from such situations as the articular cavities, as has been proved by experiment.

Another result of massage is the diminution of pain. This may be partly due to the removal of pressure from the nerves, but it is also certain that after massage the entire sensibility of the part is reduced below normal, so that there must be some action on the nerves themselves. It may be of the nature of paralysis, or of some alteration in the equilibrium between the nervous molecules, especially after "tapotement." The motor nerves, with the muscular contractility and the tone of the blood-vessels, are also affected.

Every possible form of disease has been at one time or another treated by massage, but the most important results have been seen in traumatic joint-affections, such as bruises and sprains.

The most suitable nervous cases for treatment are peripheral neuralgiæ, especially those characterized by injection and a varicose condition of the vessels of the neurilemma. Sciatica has been the neuralgia which has most repaid this treatment, but in other forms also a good result has been obtained. Articular neuroses have been remarkably improved under this treatment, but it is not to be forgotten that they are by some considered to be hysterical in nature. The other neuroses which have been treated successfully by different practioners are the minor forms of chorea, writer's cramp, hypochondria, and hysteria, infantile paralysis, and hemiplegia after apoplexy. It is particularly useful in atony of the stomach and intestines, and even volvulus has been cured by its use. It has, finally, been employed for relieving the brain of blood; and in one case of a soldier, treated by Herr Gerst, convulsions arising from nephritis were cured in four sittings in one day.

### Reflex Nervous Influence.

It has oftentimes been cast up to physicians by those who ought to know better that the mysterious and ill-defined influence of "reflex action," is utilized as a shield to cover ignorance, and as a loop-hole to crawl through and escape when confronted with a morbid condition the intimate nature and etiology of which they are unable to fathom.

That some men have availed themselves of this convenient and comprehensive term is undoubtedly true; but that such a thing as reflex action is a reality, and that it is a much more potent etiological factor of disease than is ordinarily believed, is also true.

By reflex action we mean that an impression made upon some nerve termination in one portion of the body is carried along this nerve to a centre, and from there reflected, as it were, along some other nerve to a part of the body remote from the point of first impression, at which latter point its power to disorder healthy action is made manifest, while no morbid phenomena are observable at the point from which the irritation has really arisen.

This is a plain definition of "reflex action," devoid of all technical and superfluous words; and that diseased conditions frequently have such origin, no one of experience will deny.

But the general practitioner, we fear, does not take this factor sufficiently into consideration in the formation of his opinion of the cause of disease, and since, therefore, his remedies are directed rather to the effect than to the cause of the effect, he is met oftentimes with failure, when, did he but realize the actual influence of reflex action, and look to the proper point for his cause, and guide his therapeutics accordingly, he would have much better results.

The great influence of reflex action was never more forcibly illustrated than in two cases reported by Dr. Formento, in the *New Orleans Medical and Surgical Journal* for October, 1883. In the one case a woman of good constitution, with menstruation regular but slightly painful, was married. The catamenia not occurring after marriage she was supposed to be pregnant, and additional support was given to this idea by such symptoms as occasional vomiting, irritable disposition, capricious appetite, etc.

In a couple of months, however, menstruation was re-established, but the other symptoms grew gradually worse in spite of the greatest variety of treatment.

An examination revealed an abnormal sensibility of the external organs, amounting almost to vaginismus; a narrow constricted vigina, a hard conical cervix, external or so small that the smallest sound could not pass. The cervical canal was incised laterally through its whole length, and, presto! the symptoms vanished.

After a few months they returned, and an examination revealed the fact that the canal had become again narrow; a second operation was equally successful, and so on to a third operation, after which the relief was permanent until her death from pneumonia five years after the third operation.

The second case was that of a boy, anæmic, peevish, fretful, partially paralyzed, with soft, flabby, ill-developed muscles, and all due to reflex action from the irritation of an elongated, narrow, adherent prepuce, all of which symptoms immediately yielded to circumcision. In connection with this subject, Dr. D. T. Smith, of New Orleans, says: "I am satisfied that a very large porportion of curable diseases is produced by reflex disturbance and impairment of the vitality of internal parts, caused by injuries to the terminal filaments of the nerves distributed to the skin," and in this way, rather than by the arrest of cutaneous excretion, would he explain the phenomena of the action of cold on the surface.

So much reliance does he place on the power of reflex action, that he inclines to believe that in the treatment of continued fevers, when there is a coated condition of the tongue, it would be well to coagulate the epithelium of the tongue with such an agent as Monsel's salt, and peel it off, thus making more available the reflex properties of the nerves of taste.

So, also, in the *Boston Medical and Surgical Journal*, September 27, 1883, Dr. James O. Whitney calls the attention of the profession to the following conditions frequently observed in young children: Week gait, palsy of legs, convulsions, fretting, crying, restlessness at night, and so on, which he finds due to an adherent, inelastic prepuce, for which he recommends stretching the prepuce and exposing the glands; and if this is not sufficient, circumcision.

While the influence of reflex action has been and is admitted, yet we think that it has not received the notice, as a cause of disease, that its importance demands, and it would seem that the further pursuit of the subject would offer an interesting field of research.

We can, however, formulate this practical aphorism: "When morbid conditions will not yield to appropriate treatment, look elsewhere than at the seat of manifestations for the cause, and in many cases you will wrest success from apparent failure."

